# Temporal changes in soil carbon and nitrogen in response to grazing management and vegetation cover in south-eastern Australia

**DOI:** 10.1371/journal.pone.0342006

**Published:** 2026-02-06

**Authors:** David B. Lindenmayer, Daniel Florance, Benjamin Scheele, Elle Bowd, Craig Strong, Andrew Macintosh, Maldwyn John Evans

**Affiliations:** 1 Fenner School of Environment and Society, The Australian National University, Canberra, ACT, Australia; 2 Law School, The Australian National University, Canberra, ACT, Australia; University of Eldoret, KENYA

## Abstract

Maintaining appropriate levels of carbon and nitrogen in soils is critical to the maintenance of productivity in agricultural systems. However, results vary from studies on the influence of land management, such as livestock grazing, on soil carbon and soil nitrogen. A large-scale study was implemented to quantify relationships between soil carbon, nitrogen, carbon:nitrogen ratio (C:N ratio), grazing regimes, and vegetation cover at sites on farms in south-eastern Australia, sampled in 2011 and 2022. Three grazing regimes were examined: total livestock exclusion, rotational grazing (limited duration grazing up to 45 days annually), and (continuous) set stocking rate grazing. Statistically modelled mean values for soil carbon (2011: 3%, 2022: 3.73%), nitrogen (2011: 0.21%, 2022: 0.34%), and C:N ratio (2011: 13.9, 2022: 14.3) were greater in 2022 than 2011. Soil carbon and nitrogen were greater in 2022 than 2011 in continuous grazing sites, with less pronounced time period differences in grazing exclusion and rotational sites. The C:N ratio was significantly greater in 2022 than 2011 in grazing exclusion sites (2011: 13.73, 2022: 14.58) and rotational grazing sites (2011: 13.87, 2022: 14.49), but less in 2022 (13.59) relative to 2011 in continuous grazing sites (14.31). There were inconsistent (sometimes positive, sometimes negative) empirical relationships between grazing regimes and vegetation measures as well as relationships between vegetation measures and soil carbon, soil nitrogen, and C:N ratio. Structural equation modelling (SEM) revealed limited evidence for soil carbon changes in response to vegetation attributes impacted by grazing regimes. Lower values of soil nitrogen and higher values for the C:N ratio at grazing exclusion sites were mediated by an increase in sapling abundance. SEM also identified an influence of rainfall on vegetation attributes, some of which were associated with soil properties.

## Introduction

Agriculture is a major driver of ecological degradation and species decline globally [1,2]. Many agricultural landscapes are characterised by altered vegetation communities that support limited biodiversity, degraded soils, and impaired carbon stores [3–5]. In Australia, 55% of the continent is dedicated to agricultural production, with almost 300 million hectares of native vegetation subject to livestock grazing [6,7]. For example, since European colonisation, widespread land-clearing to facilitate agricultural production (including livestock grazing) has reduced the extent of box-gum grassy woodlands in eastern Australia by ~ 95%, which now occur predominantly as isolated remnants [8,9].

Understanding above- and below-ground ecological responses to agricultural practices, such as livestock grazing, is critical to inform future practices that aim to balance production and biodiversity values in multi-use landscapes. This includes maintaining appropriate levels of carbon and nitrogen stored in soils, which is a key part of soil health for agricultural production [10–12]. Soil nitrogen influences not only nitrogen cycling, but is also linked to assimilation of carbon and, in turn, the growth of plants [12]. Similarly, maintaining vegetation cover, vegetation structure, and plant species composition is important for biodiversity, and critical to support livestock production [13]. The influence of livestock grazing on soils may be highly variable [reviewed by 10,14,15] and remains controversial [16–18]. For instance, domestic livestock grazing can influence nutrient cycles in agro-ecosystems by producing nutrient inputs (e.g., urine and faeces), consuming and defoliating biomass (which may result in a decline in productivity or a compensatory increase in growth), and modifying soil structure (e.g., compaction) [19,20], which may impede plant regeneration and further alter nutrient availability [21]. Livestock grazing can also alter the structure and composition of vegetation directly through the consumption of biomass, which can alter fuel loads for fires [22], reduce native diversity (especially grazing intolerant species) [23], and increase weed invasion [24]. Plant-soil interactions, whereby plants and soil interact to influence one another, represent another mechanism through which management practices may alter the structure and function of ecosystems [25]. For instance, plants can influence soils through the input of organic matter and chemical compounds by altering micro-climatic conditions (light availability and temperature), and by modifying below-ground microbial communities, which regulate nutrient cycling and plant growth [26,27]. Plant communities may also be shaped by livestock-associated increases in soil nutrient loads and, for example, increases in the abundance of invasive species [28,29].

Some studies have found positive relationships between grazing control (such as complete grazing exclusion) and increased soil carbon in grassland ecosystems [30,31], whereas others have documented negative relationships [32], and yet others are characterised by muted or neutral relationships [33–38]. Such variation in results appears to be somewhat context specific [*sensu* 39] and linked to (among other factors) whether native or domestic herbivores are being studied, the intensity and frequency of grazing (i.e., whether grazing occurs with or without grazing rest periods) [10], if there is some level of grazing [40] versus complete grazing exclusion [18,41], and soil nutrient levels at the start of a study [42]. Subsequently, McDonald et al. [35, p. 1] concluded that: “…..*there is a lack of evidence in Australia that grazing management directly increases soil carbon*”.

Better understanding of the influence of grazing management on vegetation and soil nutrient cycling, including carbon and nitrogen (and their interactions), is dependent on robust, replicated long-term studies [40], especially those where sites are resampled over periods exceeding a decade or longer [43,44]. Some studies are cross-sectional investigations with a response measured only once, especially for soils [18,43]. Other investigations contrast levels of vegetation and soil properties in ungrazed versus grazed areas but sometimes do not examine variations in grazing regimes (e.g., rotational grazing versus continuous grazing) [but see for example 14,18], or consider interactions between plants and soil [see 45,46]. However, evaluating differences in above- and below-ground ecological responses to different grazing practices is critical to inform future management activities to balance biodiversity and production values [47]. This is especially pertinent in threatened remnant ecosystems that occur in highly modified and fragmented landscapes [46].

Here, a large-scale investigation in south-eastern Australia is described that had an overarching objective of quantifying temporal differences between soil carbon and soil nitrogen in response to grazing management regimes and vegetation cover. Sites were sampled in 2011 and then again in 2022 and were subject to one of the following three grazing regimes: total livestock grazing exclusion, rotational livestock grazing, and conventional (continuous) livestock grazing. The study sought to answer five inter-related questions.

### Q1. Were there differences in levels of soil carbon and soil nitrogen between the initial sampling in 2011 and second sampling in 2022?

At the outset of this study, it was hypothesized there would be large differences in soil carbon and soil nitrogen between 2011 and 2022. Importantly, the grazing regime at each site (i.e., as assigned to one of the three broad categories in the study) has remained consistent over time, enabling a robust test of time period differences against a null model of no time period effects. Time period differences in soil carbon and soil nitrogen (if present) may have been associated with climate drivers [14] such as rainfall [38]. South-eastern Australia experienced a rare triple La Nina event that lasted from 2020 to the end of our resampling period in 2022, with well above average rainfall [48] (see S1 Fig). There are well-documented relationships between precipitation, vegetation, microbial activity, and soil carbon in some temperate grassland-dominated ecosystems [reviewed by 10].

### Q2. Were differences in soil carbon and soil nitrogen between 2011 and 2022 associated with grazing management?

Having tested for time period differences in Question 1, it was then appropriate to determine whether any time period differences were associated with the livestock grazing regime. This was an important test because there have been contrasting results between different studies of grazing effects on soil carbon and nitrogen [e.g., 18,35,38]. At the outset of this study, it was hypothesized there would be a time period X grazing regime interaction with values for soil carbon being: **(1)** comparatively lower in areas subject to continuous grazing, in line with the results of a global analysis of intensive grazing effects [49], including a review of studies in parts of Australia [50], and **(2)** higher in areas subject to limited grazing pressure (here rotational grazing), and where there had been complete exclusion of domestic livestock grazing [41].

The two preceding questions focused on below-ground responses. The following three questions quantified below-ground and above-ground (vegetation) responses to grazing regimes and their interactions with time period.

### Q3. Are there relationships between grazing regimes and vegetation characteristics?

It was hypothesized that there would be differences in vegetation cover and structure on sites subject to different grazing regimes. For example, previous studies have identified positive associations between rotational grazing and complete livestock grazing exclusion and sapling count and stems *<* 50 cm [51,52]. This is similar to other investigations of grazing regime impacts on vegetation [53,54].

### Q4. What are the relationships between vegetation cover, soil carbon, and soil nitrogen?

At the outset of this investigation, it was hypothesized that sites with greater levels of tree cover and other forms of vegetation, such as ground cover, would be characterised by higher values for soil carbon and soil nitrogen. It was also anticipated there would be differences in soil carbon and soil nitrogen between 2011 and 2022 as a result of changes in vegetation cover and growth, similar to those associated with rainfall (see Q1), or grazing exclusion and rotational grazing (Q3). Earlier studies have shown that an increase in tree diversity and/or the presence of larger trees can boost levels of soil carbon and nitrogen [55–58]. This is likely explained by a combination of biological processes, which regulate nutrient inputs and outputs that vary with proximity to trees (e.g., root biomass, litter quantity and soil structure), and the concentration of animals in these locations that deposit nutrients.

### Q5. What are the impacts of grazing regimes on above-ground vegetation and, in turn, levels of carbon and nitrogen in soils?

The four preceding questions focused on quantifying associations between soil carbon, soil nitrogen, livestock grazing regime, and vegetation structure. In Question 5, structural equation modelling was used to explore inter-relationships between time, grazing regime, vegetation attributes, and soil measures, and test whether changes in soil carbon and soil nitrogen were mediated through the impact of grazing on vegetation or other factors (e.g., rainfall).

## Methods

### Study area and study design

**Fig 1:**
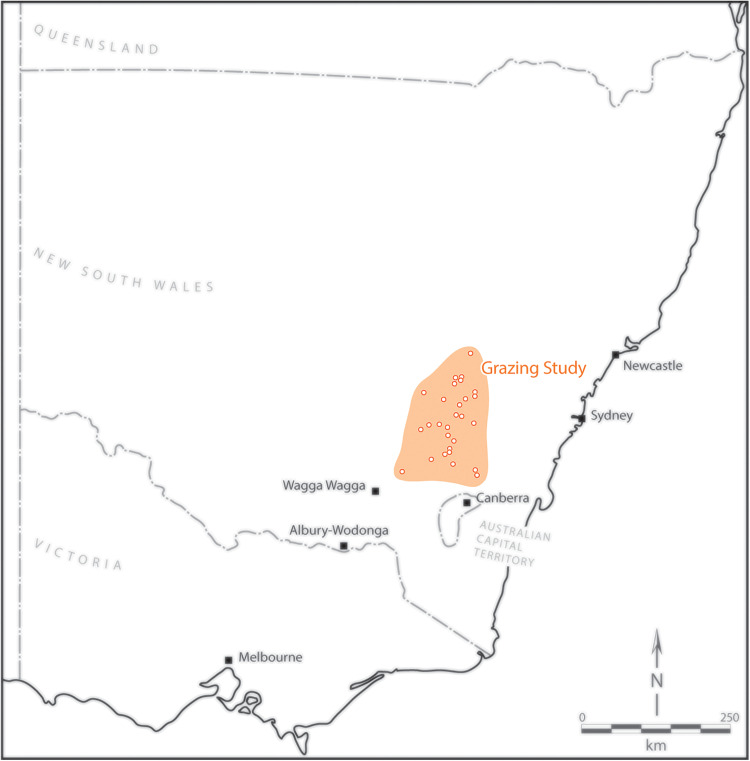
Map with location of the study region in the temperate woodlands of the Central Tablelands of New South Wales (south-eastern Australia). Field sites (shown as open circles) and the broad locations where the grazing regime and vegetation cover study was completed.

This study was located on farms distributed across a 150 km x 250 km area, approximately 100 km east to west, and 150 km north to south, in south-central New South Wales, Australia [59] (Fig 1). Geology in the region varied from mafic volcanic geology to felsic and sedimentary rocks [59,60]. Annual average rainfall in the area is approximately 625 mm with mean minimum and maximum temperatures ranging from 17^o^ - 31 °C in summer (December-February) and 8^o^ - 19 °C in winter (May-August). Extreme temperatures can be as low as −5 °C to above 40 °C.

Cropping and livestock grazing by sheep and cattle are the dominant land uses on the properties targeted in this investigation [59,61]. All farms selected for study supported woodland remnants of the Critically Endangered Box Gum Grassy Woodland community characterised by a discontinuous (20–50% cover) overstorey, dominated or co-dominated by Yellow Box (*Eucalyptus melliodora* A.Cunn. ex Schauer), White Box (*Eucalyptus albens* Benth.), Blakely’s Red Gum (*Eucalyptus blakelyi* Maiden), or Grey Box (*Eucalyptus macrocarpa* Maiden) [51]. The understorey of intact remnants supported a diverse assemblage of tussock grasses, herbs, and patchy (< 30%) shrub cover [51].

In 2010–2011, 82 permanent field sites were established on 29 farms [59] (Fig 1). These farms were involved in an agri-environment scheme that aimed to improve biodiversity conservation by paying farmers to modify grazing regimes [62]. Each of the 82 field sites comprised a 200 m long by 50 m wide (1 ha) transect and was located in a paddock covering tens of hectares and subject to one of three different grazing regimes. These were: **(1)** complete livestock grazing exclusion, with paddocks fenced to preclude access by cattle and sheep (20 sites), **(2)** continuous set stocking grazing (i.e., livestock allowed to graze sites year-round) (11 sites), and **(3)** rotational grazing, where grazing was of limited duration (totalling a maximum of 45 grazing days per site per year) with significant rest periods with no grazing (51 sites) [21,59]. Notably, the results of earlier studies on bird biodiversity and vegetation cover have indicated that sites subject to the three broad grazing regimes examined here were characterised by marked differences in the extent of native ground cover, the amount of sapling regeneration, and other vegetation attributes [51,63]. Cropping was not conducted in any of the field sites. Data on livestock stocking rates were not available as the landholders in the study were unable to consistently and reliably complete grazing diaries that had been issued to them. Although grazing diaries were not completed, there was a consensus between researchers and each landholder that each farm practiced the specific grazing treatment over the duration of the study.

In 2022, 50 of the 82 sites original sampled in 2011 (on 18 farms) were resampled. It was not possible to resample 32 of our original sites because of changes in land ownership, COVID restrictions, and/or heavy rain and flooding, that limited access to some farms. The 50 sites sampled in this study encompassed 14 sites with complete livestock grazing exclusion, nine sites subject to continuous livestock grazing, and 27 rotational grazing sites.

At the commencement of this study, the complete livestock grazing exclusion sites [see 59] were established using standard rural fencing to exclude cattle and sheep, but not the Eastern Grey Kangaroo (*Macropus giganteus*) and the introduced European Rabbit (*Oryctolagus cuniculus*). Sites subject to rotational grazing had been established 5–10 years prior to the beginning of our investigation. Continuous set stocking grazing is the conventional kind of grazing in temperate woodland environments [7] where livestock have long-term access (i.e., typically more than 180 days per annum). This practice may have been undertaken for up to a century (and sometimes longer) on sites prior to the commencement of this study [7,64,65]. Therefore, this investigation was underpinned by a quasi-experimental design [*sensu* 66] as specific before study data on grazing management practices were unavailable for conventional grazing and rotational grazing sites (see below).

### Soil sampling and laboratory analyses

Soils were collected between August and December in 2011 [59] and again in September to December 2022. At each site and during both survey periods, soil sampling was completed at 12 equally spaced points along a 200 m transect. Samples were collected using mechanical coring to a depth of 10 cm with the top 0–5 cm separated from the bottom 5–10 cm for independent analysis. Soil samples collected at each transect were bulked into sets of four and then air dried for subsequent laboratory analysis. This gave a total of three composite samples per transect for each soil depth. The same person (DF) was responsible for directing and conducting field surveys in both sampling periods.

Soil samples were crushed to reduce aggregation and particulate matter > 2 mm was removed. Samples were tested for inorganic C using hydrochloric acid [67] (Method 19D1), with no samples requiring pre-treatment for inorganic C prior to total C analysis. Total soil carbon and nitrogen concentration were assessed on finely ground soil through dry combustion using an Elementar Vario Max CNS analyser [59]. Results are reported as total C and total N percent.

Initial work in 2011 produced data on total phosphorus, total nitrogen, total carbon, and soil bulk density. However, a major change in program direction and associated project budget cuts by the Australian Government meant it was possible to complete only a smaller set of soil measures in 2022. Given this, for the purposes of the work reported here, the focus was on three response variables; total soil carbon, total soil nitrogen, and the carbon:nitrogen ratio, the differences in these values between 2011 and 2022, as well as in response to grazing regime, vegetation cover, and environmental attributes.

### Vegetation surveys, and other covariates for use in the construction of statistical models

Vegetation surveys were completed at each site between January and May in each year between 2011–2016. A further round of vegetation surveys were completed in March-April 2021. A major program change that led to severe project budget cuts by the Australian Government, coupled with restricted access to farms due to the COVID pandemic and local flooding, meant that vegetation surveys could not be conducted between 2017 and 2020.

Percentage cover for ground layer plants was measured across two point-intercept transects (50 m length) along the 200 m soil transect in each site, recording exotic and native species. Mean total cover for each 200 m x 50 m plot was determined using the spring seasonal Landsat fractional cover estimates from VegMachine [68] for each site in 2011 and 2022. The number of saplings (eucalypts < 5 cm in diameter, *Acacia* spp. plants < 2 cm in diameter measured at breast height) was also recorded, as well as the number and diameter at breast height of larger stems (> 2 m in height) in two corresponding 50 x 20 m sub-plots. Biomass of ground cover plants in each plot was measured using a rising platefall meter (Jenquip https://www.jenquip.nz/plate-meters).

Rainfall was estimated at the farm level for use as a covariate in statistical analysis using VegMachine [68] (see S1 Fig). Mean monthly rainfall was calculated for 2009–2011, and 2020–2022. Values for a topographical wetness index (TWI) were derived for each of our field sites. To do this, a digital elevation model was used at a resolution of 20 m and a buffer of 500 m around the midpoint of each site [69].

Broad geology (mafic, felsic, felsic-sedimentary, or complex) was classified at each site using the eSPADE web application developed by the New South Wales Government Department of Planning, Industry and Environment. Within eSPADE, the Surface Geology of Australia 1:1 million scale dataset 2012 edition was used [60]. Values for the latitude and longitude of each site were used in eSPADE in conjunction with Google Maps to ensure the location of each site was identified correctly.

## Statistical analysis

To test for associations between grazing regime and differences in soil carbon and nitrogen in 2011 versus 2022, a series of linear and generalised linear mixed models (LMMs, GLMMs) were fitted using the ‘glmmTMB’ [70] package in R [71]. Linear and generalised linear mixed models were used rather than standard ANOVA because the data have a hierarchical, repeated-measures structure (the same sites were sampled in both 2011 and 2022, and sites are nested within farms). Mixed models account for this non-independence through random effects (Site and Farm) while testing fixed effects of Period, Treatment, and their interaction. A depth factor variable (0–5 cm, 5–10 cm) was included in all models to account for the differences in response variables at these depths. Statistical significance was assessed using the p-values generated from the likelihood ratio tests inherent to these mixed-effects models.

### Were there differences in levels of soil carbon and soil nitrogen between the initial sampling in 2011 and second sampling in 2022?

To answer this question, GLMMs were fitted to test the effect of the *time period* factor variable (2011 vs 2022) on response variables (total carbon (%), total nitrogen (%), and the carbon:nitrogen ratio [C:N ratio]):


$${\gamma_{i}\;\sim \;\beta }_{\text{0}}{+\;\beta_{\text{1}}Y}_{y}+{{\beta_{\text{2}}D}_{s}+f}_{i}+\;i_{i},$$

where $\gamma_{i}$ is the predicted response of a given response variable at site *i,* $\beta_{\text{0}}$ is the intercept, $\beta_{\text{1}}$ and $\beta_{\text{2}\;}$ are the associated regression coefficients representing the linear effects of *time period* ($Y_{y}$) and *depth* ($D_{s}$) at year *y* and sample *s*. Farm-$\;(f_{i})$ and site- $\left(i_{i}\right)$ level random effects were included to allow for dependence of repeated measures at the same sites within and between years, and farm management similarities within a given farm. A beta error distribution was assumed for the total carbon and total nitrogen response models, where values ranged between zero and one (equivalent to 0% and 100%). A Gaussian error distribution was assumed for the C:N ratio model. Models were fitted using the ‘glmmTMB’ [70] package in R [71].

### Q2. Were differences in soil carbon and soil nitrogen between 2011 and 2022 associated with grazing management?

To answer this question, GLMMs were fitted to test the fixed and interactive effects of *time period* and *treatment* on response variables:


$${\gamma_{i,y}\;\sim \;\beta }_{\text{0}}{+\;\beta_{\text{1}}Y}_{y}{\;+\;\beta_{\text{2}}GR}_{i}{+\;\beta_{\text{3}}D}_{s}{{\;+\;\beta_{\text{4}}Y_{y}GR}_{i\;}+\;f}_{i}+\;i_{i},$$

where $\gamma_{i,y}$ is the predicted response of a given response variable at site *i* in year *y,* $\beta_{\text{0}}$ is the intercept, $\beta_{\text{1}}$ to $\beta_{\text{4}\;}$ are the associated regression coefficients representing the linear effects of *year* ($Y_{y}$), *grazing regime* ${(GR}_{i})$, and *depth* ($D_{s}$), and the interactive effect of *time period* and *grazing regime* ${(Y}_{y}{GR}_{i}$ at site *i*, year *y*, and sample *s*. The error distributions and random effects were the same as in Q1. Models were again fitted using the ‘glmmTMB’ [70] package in R [71]. An interactive effect between time period and treatment was interpreted as indicative of grazing treatment effects between 2011 and 2022.

### Q3. Are there relationships between grazing regimes and vegetation measures?

To answer this question, multiple GLMMs were fitted to test the fixed and interactive effects of *time period* and *treatment* on the vegetation variables (*fractional cover, biomass, saplings, ground cover (exotic), ground cover (native), stems 5–50 cm, stems > 50 cm*). Using *biomass* as an example response:


$${\delta_{i}\;\sim \;\beta }_{\text{0}}{+\;\beta_{\text{1}}Y}_{y}{\;+\;\beta_{\text{2}}GR}_{i}{{\;+\;\beta_{\text{3}}Y_{y}G}_{i\;}+\;f}_{i}+\;i_{i},$$

where $\delta_{i}$ is the predicted *biomass* at site *i*, $\beta_{\text{0}}$ is the intercept, $\beta_{\text{1}}$ to $\beta_{\text{3}\;}$ are the associated regression coefficients representing the linear effects of *year* ($Y_{y}$), *grazing regime* ${(GR}_{i})$, and the interactive effect of *time period* and *grazing regime* $(Y_{y}{GR}_{i}$ at year y and sample *s*. A Gaussian error distribution was assumed for *biomass*, *ground cover (exotic)*, and *ground cover (native)*, a Poisson error distribution for *saplings*, *stems 5–50 cm*, and *stems > 50 cm*, and a Beta error distribution for *fractional cover.* Models were fitted using the ‘glmmTMB’[70] package in R [71].

### Q4. What are the relationships between vegetation cover, soil carbon and soil nitrogen?

To answer Question 4, a step-wise model selection approach was employed using Akaike’s Information Criterion for small samples sizes (AICc) [72]. For the carbon, nitrogen, and C:N ratio response variables, sets of models were fitted considering all predictor variables in Table 1, employing a stepwise model-selection procedure using AICc [72] to select the most parsimonious model from a candidate set. The full model included fixed and interactive effects:

**Table 1:** Variables used in statistical analyses. Columns Q1 to Q4 represent how each variable was used in our questions. In these columns R represents as a response variable, P as a predictor variable, and RI as a random intercept.

Name	Symbol	Q1	Q2	Q3	Q4	Q5	Variable type	Brief summary
Total soil carbon		R	R		R	R	Continuous (proportion) Logit transformed for Q5.	Carbon as a proportion of the sample.
Total soil nitrogen		R	R		R	R	Continuous (proportion) Logit transformed for Q5.	Nitrogen as a proportion of the sample.
C:N ratio		R	R		R	R	Continuous (ratio)	The ratio between carbon and nitrogen.
Time period	$Y_{y}$	P	P	P	P	P	Factor with two levels: • 2011 • 2022 Dummy variable created for Q5.	The year in which soil samples were collected. Dummy variable created for Q5.
Soil depth	$D_{s}$	P	P		P		Factor with two levels: • 0–5 cm • 5–10 cm	The depth of the soil sample taken.
Grazing regime	${\;GR}_{i}$		P	P		P	Factor with three levels: • Conventional continuous grazing • Rotational • Exclusion Dummy variables created for Q5.	Dummy variables created for Q5.
Fractional cover	${FC}_{i,y}$			R	P	R,P	Continuous (proportion). Logit transformed for Q5.	VegMachine was used which is an online tool that uses satellite imagery to summarise change in Australia’s landscape [68]. Mean values for each site were calculated in 2011 and 2022. Note that some months yielded no data and these rows were removed.
Biomass	$B_{i,y}$			R	P	R,P	Continuous	Rising platefall meter used to measure ground cover biomass (Jenquip https://www.jenquip.nz/plate-meters).
Saplings	${\;S}_{i,y}$			R	P	R,P	Continuous (count)	Number of saplings (eucalypts < 5 cm, Acacias < 1 cm in 50 x 20 m plot).
Ground cover (native)	${\;CN}_{i,y}$			R	P	R,P	Continuous	Percentage native ground covers using point-intercept transect – measured at 1m intervals along 50 m transect (2x transects per site).
Ground cover (exotic)	${\;CE}_{i,y}$			R	P	R,P	Continuous	Percentage exotic ground covers using point-intercept transect – measured at 1m intervals along 50 m transect (2x transects per site).
Stems 5–50 cm	${\;STS}_{i,y}$			R	P	R,P	Continuous	Number of stems 5–50 cm Diameter at Breast Height (DBH) within 50 x 20 m plot (2x plots per site).
Stems > 50 cm	${\;STL}_{i,y}$			R	P	R,P	Continuous	Number of stems > 50 cm DBH within 50 x 20 m plot (2x plots per site)
Geology	${\;G}_{i}$				P		Factor with four levels: • Mafic • Felsic • Felsic-sedimentary • Complex	Broad geology class at each site was classified using the eSPADE web application developed by the New South Wales Government Department of Planning, Industry and Environment. Within eSPADE, the Surface Geology of Australia 1:1 million scale dataset 2012 edition was used. Values for the latitude and longitude of sites was used in eSPADE in conjunction with Google maps to ensure the location of each site was identified correctly.
Topographical Wetness Index (TWI)	${\;TWI}_{i}$				P		Continuous (proportion)	Values for a topographical wetness index (TWI) were derived from a digital elevation model at a resolution of 20 m and a buffer of 500 m around the midpoint of each site [69].
Rainfall	$R_{i,y}$				P		Continuous	Gridded rainfall collected at the farm level using VegMachine [68]. Mean monthly rainfall was calculated for 2009–2011 and 2020–2022.
Farm	$f_{i}$	RI	RI	RI	RI	RI	Factor with 25 levels.	The farms where the sites were located. 29 farms in 2011, 18 farms in 2022.
Site	$S_{i}$	RI	RI	RI	RI	RI	Factor with 83 levels.	The sites where soil samples were collected. 82 in 2011, 50 in 2022.


$$\begin{array}{cc} \gamma_{i,y} & \sim \beta_{0}+\beta_{1}Y_{y}+\beta_{2}FC_{i,y}+\beta_{3}B_{i,y}+\beta_{4}CN_{i,y}\\ & \;+\beta_{5}CE_{i,y}+\beta_{6}S_{i,y}+\beta_{7}STS_{i,y}+\beta_{8}STL_{i,y}\\ & \;+\beta_{9}D_{i,y,s}+\beta_{10}G_{i}+\beta_{11}TWI_{i}+\beta_{12}Y_{t}FC_{i,y}\\ & \;+\beta_{13}Y_{t}P_{i,y}+\beta_{14}Y_{t}CN_{i,y}+\beta_{15}Y_{t}CE_{i,y}+\beta_{16}Y_{y}S_{i,y}\\ & \;+\beta_{17}Y_{y}STS_{i,y}+\beta_{18}Y_{y}STL_{i,y}+f_{i}+i_{i}, \end{array}$$

where $\gamma_{y}$ is the predicted response of a given response variable at site *i* in year *y,* $\beta_{\text{0}}$ is the intercept, $\beta_{\text{1}}$ to $\beta_{\text{1}\text{9}\;}$ are the associated regression coefficients representing the linear and interactive effects of the predictors. In addition to quantifying the effects on soil carbon, soil nitrogen and C:N ratio of time and the vegetation variables, the influence of key site characteristics also was determined such as the underlying geology $\left(G_{i}\right)$, and the topographical control of hydrological processes $\left({TWI}_{i}\right)$ [73]. The use of GLMMs accommodated the different number of sites in 2011 and 2022.

The same error distributions as in Q1 were assumed, using the ‘buildmer’ package [74] to conduct stepwise model selection using models fitted using the ‘glmmTMB’ [70] package in R [71]. An interactive effect between time period and a vegetation variable predictor was interpreted as that vegetation variable predicting a given soil response over the time of the study (between 2011 and 2022).

### Q5. What are the impacts of grazing regimes on above-ground vegetation and, in turn, levels of carbon and nitrogen in soils?

To answer Q5 associated with grazing regimes, soil carbon, nitrogen and vegetation (and their interactions), a structural equation model (SEM) [75] was implemented using the ‘piecewiseSEM’ package [76] in R [71]. A SEM was employed to test multivariate causal pathways linking grazing management, vegetation characteristics, and soil carbon and soil nitrogen. SEM was selected because it allows simultaneous testing of multiple dependent variables and direct/indirect effects, providing a more complete picture of how grazing influences soil carbon and soil nitrogen through vegetation-mediated pathways. The SEM explicitly tested the impacts of the grazing regimes on vegetation metrics, including the interaction between the grazing regime and time period, as well as the cascading impacts of changes in rainfall (see S1 Fig) and vegetation changes on soil carbon and soil nitrogen. The SEM was restricted to these relationships, assuming that other associations between variables had been explored in Q1 and Q2. This approach also avoided over-parametrising the model, potentially leading to model-fitting issues. For the purposes of this analysis, several variables were transformed for use in linear models with assumed Gaussian error distributions (Table 1). All the vegetation variables and rainfall were scaled to have a mean of 0 and standard deviation of 1. The SEM parametrisation was as follows:


$${SC}_{i,y}\;\sim \;\;{FC}_{i,y}{+\;{\;B}_{i,y}{+\;CN}_{i,y}\;{+\;CE}_{i,y}+\;S}_{i,y}+{\;STS}_{i,y}{+\;STL}_{i,y}+\;{GR}_{i}+\;Y_{y}+{GR}_{i}Y_{y}+R_{i,y},$$ (1)

$${SN}_{i,y}\;\sim \;\;{FC}_{i,y}{+\;{\;B}_{i,y}{+\;CN}_{i,y}\;{+\;CE}_{i,y}+\;S}_{i,y}+{\;STS}_{i,y}{+\;STL}_{i,y}+{GR}_{i}+\;Y_{y}+{GR}_{i}Y_{y}+R_{i,y},$$ (2)

$${SCNR}_{i,y}\;\sim \;\;{FC}_{i,y}{+\;{\;B}_{i,y}{+\;CN}_{i,y}\;{+\;CE}_{i,y}+\;S}_{i,y}+{\;STS}_{i,y}{+\;STL}_{i,y}+{GR}_{i}+\;Y_{y}+{GR}_{i}Y_{y}+R_{i,y},$$ (3)

$${FC}_{i,y}\;\sim \;\;{GR}_{i}+\;Y_{y}+{GR}_{i}Y_{y},$$ (4)

$$B_{i,y}\;\sim \;\;{GR}_{i}+\;Y_{y}+{GR}_{i}Y_{y},$$ (5)

$${CN}_{i,y}\;\;\sim \;\;{GR}_{i}+\;Y_{y}+{GR}_{i}Y_{y},$$ (6)

$${CE}_{i,y}\;\sim \;\;{GR}_{i}+\;Y_{y}+{GR}_{i}Y_{y},$$ (7)

$$S_{i,y}\;\sim \;\;{GR}_{i}+\;Y_{y}+{GR}_{i}Y_{y},$$ (8)

$${STS}_{i,y}\;\sim \;\;{GR}_{i}+\;Y_{y}+{GR}_{i}Y_{y},$$ (9)

$${STL}_{i,y}\;\sim \;\;{GR}_{i}+\;Y_{y}+{GR}_{i}Y_{y}.$$ (10)

$$R_{i,y}\;\sim \;{GR}_{i}+\;Y_{y}+{GR}_{i}Y_{y}.$$ (11)

To facilitate communication, coefficient symbols (e.g., β_0_ to β_n_) were not included. Each model component (1) to (10) also included random intercept effects for site and farm, as in earlier models. For the purposes of fitting this model, dummy variables were created for rotational grazing sites (Non rotational = 0, Rotational = 1) and grazing exclusion sites (Non exclusion = 0, Exclusion = 1), as well as the time period variable (2011 = 0, 2022 = 1). Therefore, in models (4) to (10), these models were specified as:


$${GR}_{i}+\;{GR}_{y}+{GR}_{i}Y_{y}=\;{GR}_{Rotational}+{GR}_{Exclusion}+Y_{\text{2}\text{0}\text{2}\text{2}}+{GR}_{Rotational}Y_{\text{2}\text{0}\text{2}\text{2}}+{GR}_{Exclusion}Y_{\text{2}\text{0}\text{2}\text{2}}.$$

This approach allowed us to assume the sites subject to continuous grazing in 2011 as the reference levels in the model components. Individual models were fitted within the SEM using the glmmTMB [70] package. To account for potential non-independence between variables that are not part of the main causal pathways, they were included as correlated errors (non-causal interactions) in the SEM. This approach controlled for correlations without implying direct causality. The model was tested using Shipley’s test of directed separation (d-sep) to evaluate the conditional independence of the variables not included in the causal paths [77].

## Results

### Q1. Were there differences in levels of soil carbon and soil nitrogen between the initial sampling in 2011 and second sampling in 2022?

**Fig 2:**
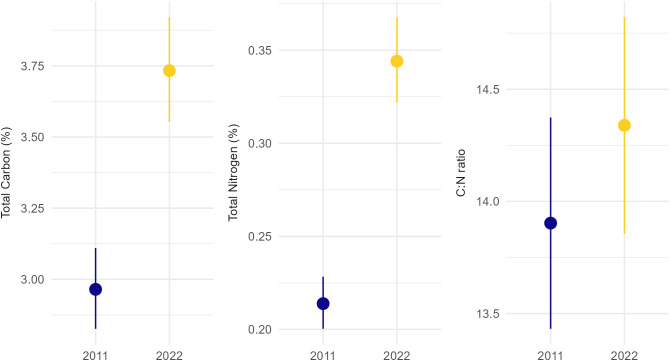
Model-predicted means and standard errors for values of Total Carbon (%), Total Nitrogen (%), and C:N ratio in 2011 and 2022 samples. For model summary tables see S1 Table.

There was strong evidence for greater levels of soil carbon (P < 0.001), soil nitrogen (P < 0.001), and the C:N ratio (P = 0.013) in 2022 relative to 2011 (Fig 2).

### Q2. Were differences in soil carbon and soil nitrogen between 2011 and 2022 associated with grazing management?

**Fig 3:**
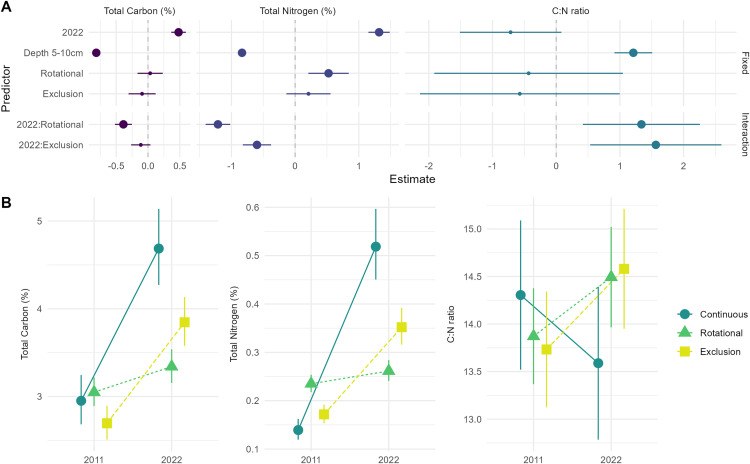
A. Effect sizes and 95% confidence intervals for models fitted in Q2. Effects were considered to be significant if their 95% confidence intervals did not cross the zero-effect line. Large points represent those significant effects. **B.** Model-predicted means and standard errors for values of Total Carbon (%), Total Nitrogen (%), and C:N ratio in the treatments in each time period. For model summary tables see S2 Table.

There was strong evidence for an interaction between grazing regime and time period for soil carbon, soil nitrogen, and the C:N ratio (Fig 3 and S2 Table). For both soil carbon and soil nitrogen, values in the continuous grazing sites were greater in 2022 than in 2011, whereas there was limited difference between 2011 and 2022 in sites subject to rotational grazing (Fig 3B and S2 Table). Values in the grazing exclusion sites were greater in 2022 relative to 2011 but significantly less so than the continuous grazing sites (Fig 3B). The analyses showed that the C:N ratio was greater in 2022 and 2011 in both the rotational grazing and grazing exclusion sites, but lower in 2022 compared to 2011 on sites subject to continuous grazing (Fig 3B).

### Q3. Are there relationships between grazing regimes and vegetation measures?

**Fig 4:**
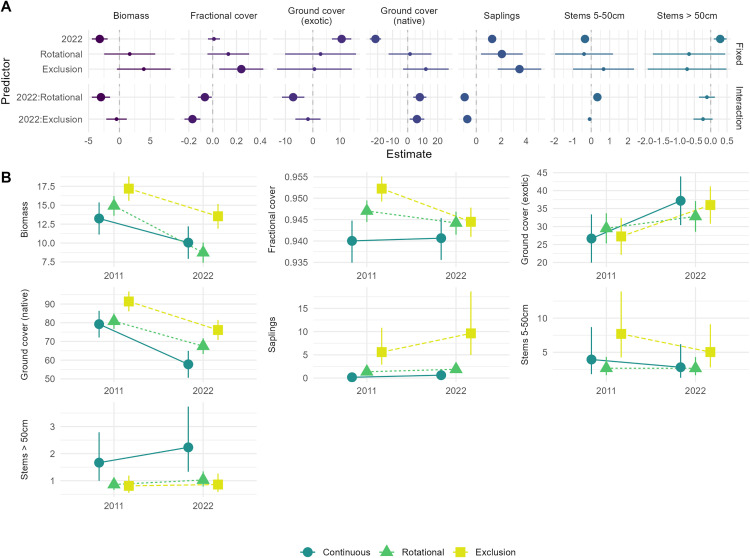
A. Effect sizes and 95% confidence intervals for models fitted in Q3. Effects were considered to be significant if their 95% confidence intervals did not cross the zero-effect line. Large points represent those significant effects. **B.** Model-predicted means and standard errors for the vegetation variables in the treatment sites and in the two time periods. For model summary see S3 Table.

There were differences among grazing regimes in vegetation measures for the two time periods (Fig 4). Fractional cover was greatest in the grazing exclusion sites, but was lower in 2022 than in 2011 (this effect was most pronounced at grazing exclusion sites). Native ground cover was lower in 2022 than in 2011 on all sites. However, sites subject to rotational grazing and grazing exclusion showed a smaller temporal difference, as evidenced by the positive interactive effects between these sites and year (Fig 4). Similarly, exotic ground cover was greater in 2022 than in 2011 across all sites, but rotational grazing sites exhibited a smaller increase. There were more saplings in rotational grazing and grazing exclusion sites compared to continuous grazing sites, but these sites exhibited a slower rate of increase over time than in continuous grazing sites (Fig 4).

### Q4. What are the relationships between vegetation cover, soil carbon, and soil nitrogen?

**Fig 5:**
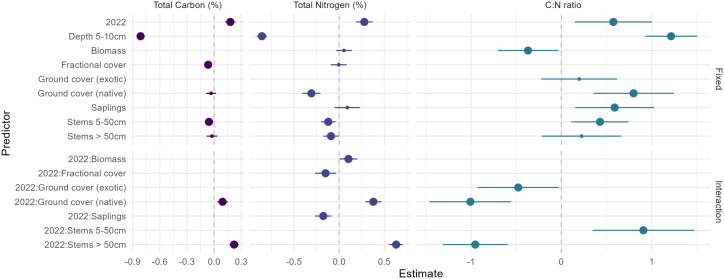
Effect sizes and 95% confidence intervals for predictor variables retained in final models for Q4. Effects were considered significant if their 95% confidence intervals did not cross the zero-effect line (large points). Only variables that improved model fit during model selection are shown; excluded variables had negligible effects or did not contribute to explaining variation in the response. For model summary tables see S4 Table.

**Fig 6:**
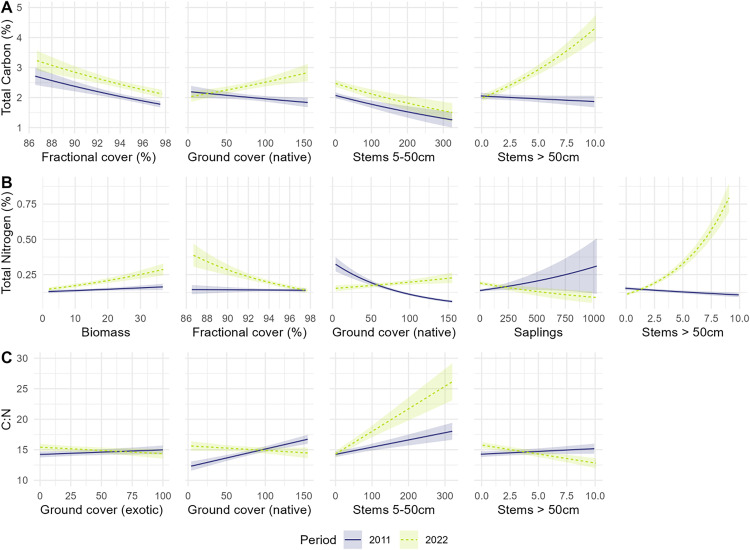
Predicted relationships between soil properties (total carbon [A], total nitrogen [B], C:N ratio [C]) and vegetation structure/cover variables retained in final models, showing interactive effects with time period. Error bands represent standard errors. Only variables that were retained during model selection and showed meaningful effects are displayed.

The best-fit model for soil carbon showed that it was negatively related to fractional cover and stems 5–50 cm (Figs 5 and 6). There also was a positive association between native ground cover and stems > 50 cm and soil carbon, but only in 2022. Soil sampled at 5–10 cm had lower levels of carbon than soil sampled at 0–5 cm.

The best-fit model for soil nitrogen indicated it was negatively associated with native ground cover in 2011 but positively associated with native ground cover in 2022 and the number of stems over 50 cm (Figs 5 and 6). Soil nitrogen was negatively associated with the number of stems 5–50 cm in both years of sampling. Soil nitrogen was also negatively associated with fractional cover and saplings, but only in 2022. In contrast, soil nitrogen was positively associated with biomass in 2022, but less so in 2011. Soil collected at 5–10 cm had lower levels of nitrogen than soil sampled at 0–5 cm (Fig 5).

The best-fit model for C:N ratio indicated that native ground cover, exotic ground cover, and stems over 50 cm were positively associated with C:N ratio in 2011 but negatively associated with it in 2022 (Figs 5 and 6). C:N ratio was negatively associated with exotic ground cover, but only in 2022. C:N ratio was also negatively associated with Biomass. Soil sampled at 5–10 cm had a higher C:N ratio than soil sampled at 0–5 cm.

### Q5. What are the impacts of grazing regimes on above-ground vegetation and, in turn, levels of carbon and nitrogen in soils?

**Fig 7:**
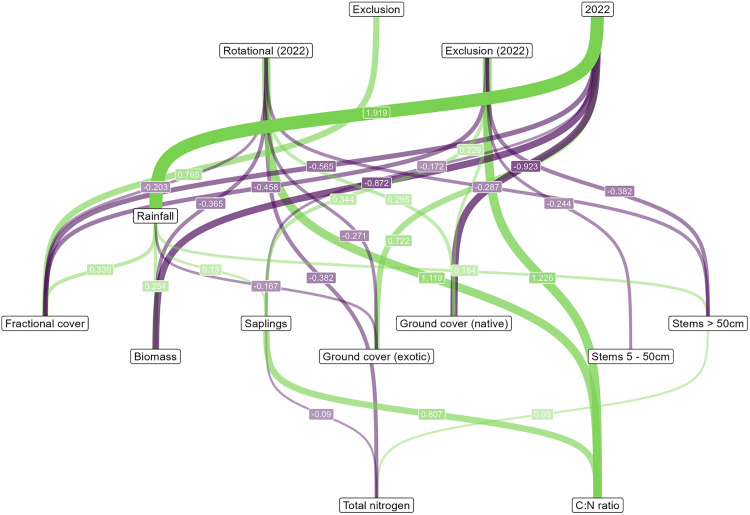
Direct acyclic graph representing the results of the SEM in Q5 (Fisher’s C = 0.881, P = 0.927, DF = 4). Only those relationships considered significant (P < 0.05) have been plotted. Effects are directed top (predictors) to bottom (responses). Green represents positive effects and purple represents negative effects. Line width and transparency represent the relative strengths of the effects. ‘Exclusion’ is the effect of exclusion sites compared to continuous grazing. ‘2022’ is the effect of time period (values in 2022 compared to those in 2011). ‘Rotational (2022)’ and ‘Exclusion (2022)’ represent the interactive effects of the Rotational grazing and Exclusion grazing sites compared to the 2011 sites. See S5 and S6 Tables for model summary tables.

There was limited evidence for changes in total carbon as a result of the grazing regime treatments impacts on vegetation (Fisher’s C = 0.881, P = 0.927, DF = 4) (Fig 7 and S5 and S6 Tables). There was, however, evidence that for all sites in 2022, there was greater rainfall than in 2011 (see also S1 Fig), which, in turn likely led to an increase in saplings and stems > 50 cm. Subsequently, increases in saplings were negatively associated with soil nitrogen, but increases in stems > 50 cm were positively associated with soil nitrogen (Fig 7).

There was strong evidence for greater values for the C:N ratio in both rotational grazing and grazing exclusion sites in 2022 versus 2011 relative to continuous grazing sites in those same two sampling periods (Fig 7). The only evidence for a vegetation-mediated effect from the grazing treatments for the C:N ratio, was that livestock grazing exclusion sites in 2022 were characterised by more saplings, which, in turn, was associated with a higher C:N ratio (Fig 7).

## Discussion

Substantial amounts of the world’s terrestrial carbon stores are in the soil [11,40]. However, large-scale and long-term studies of soil carbon and soil nitrogen are relatively rare [but see for example 44], including in response to different grazing regimes (e.g., continuous versus rotational grazing) [43]. Here, relationships were quantified between levels of soil carbon and soil nitrogen, grazing regimes, and vegetation cover (and their interactions) on the same sites measured 11 years apart. The work produced evidence of strong time period differences between 2011 and 2022 in soil carbon, soil nitrogen, and the C:N ratio, but also complex responses to grazing and vegetation cover. These and other key findings are discussed in the remainder of this paper, together with some of the potential implications of the study for vegetation and grazing regimes and land management policies.

### Q1. Were there differences in levels of soil carbon and soil nitrogen between the initial sampling in 2011 and second sampling in 2022? Q2. Were differences in soil carbon and soil nitrogen between 2011 and 2022 associated with grazing management?

There was evidence for greater values for soil carbon and soil nitrogen in 2022 than 2011 across all sites. Some effects (e.g., for soil nitrogen and C:N) (see Fig 7) appear to be broadly influenced by levels of rainfall, that were markedly lower in 2011 relative to those in 2022 (S1 Fig). As outlined further below, rainfall was strongly associated with time period differences in vegetation such as biomass, the number of stems > 50 cm, the abundance of saplings and fractional cover, some of which were associated with changes in soil properties (Fig 7).

There was strong evidence for greater values for soil carbon and soil nitrogen in the continuous grazing sites in 2022 than 2011, less pronounced time period differences in grazing exclusion sites, and little evidence of time period differences on sites subject to rotational grazing (Fig 3). These results are not consistent with the findings of global meta-analyses and other reviews [10,40,41], although some studies have found limited or no relationships between soil carbon and grazing management factors such as the grazing regime and stocking density [35,38,78].

The reasons for associations between soil carbon and soil nitrogen and grazing regime which characterised this study remain unclear. The higher values recorded for soil nitrogen in sites subject to continuous grazing relative to other treatments may be related to a combination of more exotic ground covers like exotic annual grasses, forbs (e.g., clovers) [79] and lucerne, as well as a greater number of livestock contributing more faecal matter and urine to the soil surface over a longer period compared to areas subject to rotational grazing [80].

The C:N ratio of soils is indicative of how much nitrogen can be potentially mineralised per unit of respired carbon. This measure was greater in 2022 than in 2011 for both the rotational grazing sites and grazing exclusion sites, but lower on sites subject to continuous grazing. These findings are consistent with literature from the same and similar temperate woodland ecosystems to the ones studied here, and which describe an increase in soil fertility with increasing livestock-generated degradation (as measured by decreased native plant diversity) of grasslands [57,81,82]. Moreover, these abiotic modifications encourage the establishment of nitrophilic exotic annual plants, which perpetuate highly fertile soils, with lower carbon:nitrogen values via plant-soil feedbacks [55,83,84].

### Q3. Are there relationships between grazing regimes and vegetation measures?

There was evidence of relationships between grazing regimes and measures of vegetation, but these varied between the two time periods studied. Both fractional cover and sapling abundance were lower on sites subject to continuous grazing than grazing exclusion. There was also weak evidence that native ground cover was higher overall in grazing exclusion sites. Native ground cover was lower in 2022 than in 2011 on all sites. However, sites subject to rotational grazing and grazing exclusion showed a smaller relative temporal difference, as evidenced by the positive interactive effects between these types of sites and year (Fig 4). Similarly, exotic ground cover was greater in 2022 than in 2011 across all sites, but rotational grazing sites exhibited a smaller temporal difference relative to sites subject to continuous grazing. It is possible that rotational grazing sites and grazing exclusion sites might have been buffered from the overall temporal effect of greater exotic ground cover in 2022. These findings are broadly consistent with other studies which have shown that reduced grazing pressure (corresponding to grazing exclusion and rotational grazing in this investigation) can positively affect vegetation measures [e.g., 52, reviewed by 53,54].

### Q4. What are the relationships between vegetation cover, soil carbon and soil nitrogen? Q5. What are the impacts of grazing regimes on above-ground vegetation and, in turn, levels of carbon and nitrogen in soils?

There was evidence of temporal differences in the influence of vegetation measures (as indicated by two-way interactions between vegetation and time period) on soil carbon, soil nitrogen, and the C:N ratio (Figs 5 and 6 and S2 Table). For example, there was a positive association between soil carbon and stems > 50 cm in diameter, but only in 2022. Other studies in Australian temperate woodlands and forests have shown there are higher levels of soil carbon in areas dominated by larger trees [45,58,85,86]. For example, both Prober et al. (56) and Addo-Danso [45] reported more fertile soils under trees (as reflected by an increase in total carbon and available nitrogen), relative to open areas. Bowd and Lindenmayer (55) also found a positive association between soil nitrate and available phosphorus, and an increase in the basal area of trees in box-gum grassy woodland environments. Such relationships may be explained by biological processes such as litter accumulation, deep root systems which retain nutrients [e.g., 86], and/or a concentration effect of livestock faecal inputs as domestic animals typically congregate under larger trees [80]. Similarly, Chen et al. [55] found that an increase in the diversity of trees can lead to an increase in soil carbon and soil nitrogen over decadal time scales, although other investigations have been characterised by more muted relationships [87]. Similarly, forestation of abandoned and farmed lands can have marked effects on microbial biomass carbon and nitrogen [88].

In contrast to soil associations with larger stems, there was a negative relationship between saplings, soil nitrogen and soil carbon levels, and a positive relationship with C:N ratio. This is likely explained by the ecological preferences of native plant species (including eucalypts in our study system) which are adapted to low-nutrient conditions [52,83,84]. Higher-fertility soils, which perpetuate nitrophilic exotic plant species, may also negatively affect tree regeneration through competitive exclusion [52].

Recent reviews have suggested that whilst evidence of direct relationships between grazing management and soil carbon is limited [35], grazing can alter some of the factors that influence soil carbon sequestration and storage including above and below-ground biomass [10, but see 47]. However, in this investigation, structured equation modelling which was designed to explore inter-relationships between time, grazing regime, vegetation attributes, and soil measures, revealed limited evidence that changes in vegetation in response to the grazing regimes impacted soil carbon or soil nitrogen. The only evidence for these cascading effects was shown with livestock exclusion sites in 2022 having comparatively more saplings than other sites in that year, which in turn, was negatively associated with soil nitrogen and positively associated with C:N ratio (Fig 7). The reasons for the paucity of clear inter-relationships between grazing regime, vegetation, soil carbon and soil nitrogen may be associated, in part, with variations in stocking rates and grazing duration within and between two of our grazing categories (continuous grazing and rotational grazing). Coupled with this, there may also have been impacts of herbivores other than domestic livestock in our grazing exclusion treatment. These issues are further explored in the section below on study limitations.

SEM revealed that temporal differences in rainfall (see S1 Fig) influenced vegetation attributes. There was higher rainfall in 2022 versus 2011 (S1 Fig) and this was linked to vegetation measures such as the abundance of saplings and stems > 50 cm, Fig 7). Analysis using SEM also contained evidence of associations between these vegetation measures and soil measures (C:N and soil nitrogen), which suggests possible plant-mediated effects of rainfall on soils (Fig 7). By increasing the diffusion and mass flow of nutrients, rainfall patterns can increase nutrient availability, and therefore productivity, germination, and biomass growth [89,90]. However, rainfall was not a clear driver of change in soil carbon. The influence of rainfall on diversity and productivity in grassland and woodland ecosystems, and the relative likely effects on soils, requires important consideration in restoration practice and policy [91].

### Policy and management implications

The results of this study have important implications for policies related to land management. The analysis reported here showed that time period was a major factor associated with levels of soil carbon and soil nitrogen. Time period may, in part, be a proxy for weather and climate with, as outlined above, 2022 being the third successive year of well above average rainfall in this study (see Fig 7). Notably, other studies have highlighted the dominant role of climate as opposed to grazing regimes on measures such as primary productivity [e.g., 92]. Therefore, a key issue is to consider how best to maintain carbon and nitrogen soil stocks when climate conditions change and, for example, when drier conditions (and possibly even drought) occur [93]. This highlights the importance of ensuring the establishment of long-term monitoring programs characterized by frequent and repeated measurements of soil carbon and nitrogen levels. The design of these programs also need to include external experimental controls that can help quantify not only the impacts of grazing management, but also the influence of other drivers such as vegetation cover as well as climate and weather [50].

A strength of this study was that a large number of sites were sampled on many farms. In addition, soil carbon, soil nitrogen, and vegetation attributes were measured directly at the same sites and relationships between them explored with rigorous statistical analyses. A key implication of such an approach is the potential for interactions between time, grazing management, and vegetation measures, that can help produce a more detailed mechanistic understanding of the drivers of change in soil carbon and soil nitrogen (as reflected in the SEM, see Fig 7).

### Study limitations

This study has several limitations. First, whilst the farmers in the study initially kept accurate grazing diaries for the number of livestock in particular sites and the length of grazing periods, this information was not recorded throughout the entire duration of the study as it relied on farmers goodwill to collect these data. In some cases, such as during droughts, prolonged and extensive destocking may result in continuous grazing regimes having less grazing pressure than areas subject to rotational grazing. Indeed, the rotational grazing regimes employed in our investigation appeared to be highly variable, both in terms of the number of animals being grazed and the time they were grazing in particular paddocks. Many livestock enterprises are reactive to market and seasonal conditions and may change their stocking rates accordingly. Such variation may have been a factor underpinning the marked variability in soil carbon and soil nitrogen that characterised our rotational grazing sites.

Second, the grazing exclusion treatment in this study excluded domestic livestock but did not preclude grazing and browsing by native macropods (e.g., Eastern Grey Kangaroo [*Macropus giganteus*], Euro [*Osphranter robustus*], Swamp Wallaby [*Wallabia bicolour*]) and the introduced European Rabbit (*Oryctolagus cuniculus*). These species can have significant impacts on ground cover when in large numbers [94]. However, it was too expensive to erect and maintain fences capable of preventing the access of both native and introduced feral herbivores.

A third limitation of this study was that soil sampling was confined to 0–5 cm and 5–10 cm below the ground surface. Grazing management may change the distribution in soil carbon and nitrogen deeper in the soil profile, and sampling to greater depths would therefore have been an advantage [e.g., 86]. Indeed, sampling at greater depths and measurement of bulk density would have been useful to further examine relationships between grazing management and carbon stocks. In addition, there would be value in measuring bulk density in future work related to soil carbon, soil nitrogen and livestock grazing regimes.

Fourth, the study design included pre-treatment soil carbon and soil nitrogen measures for grazing exclusion sites, but not for sites subject to rotational grazing or continuous grazing. This prevented us from implementing a conventional before/after/control/impact (BACI) design [*sensu* 95] for all three grazing regimes examined. However, such a design would be extremely difficult to implement given that widespread domestic livestock grazing has been ongoing for 150–200 years in our study region [64,65] and very few places exist without such a prolonged history of past disturbance. Hence, the quasi-experimental design [*sensu* 66] at a landscape scale that was implemented was the most appropriate for this study. We note that a robust carbon credit scheme for soil carbon would need to account for pre- and post-management interventions [96], such as shift from conventional grazing to rotational grazing or complete grazing exclusion [97] as well as appropriately matched experimental controls for management treatments. Indeed, a paucity of data on the management history of sites is a limitation of many studies of grazing management such as those on rotational grazing [98]. Finally, despite extensive sampling 11 years apart, the data gathered in this study were nevertheless limited to two points in time (2011 and 2022). Consequently, how levels of soil carbon and soil nitrogen have fluctuated during the intervening years (including during drought periods) remains unknown. More intensive repeated sampling would rectify this, but conducting annual or biannual field sampling and associated laboratory analyses was neither logistically nor financially possible in this investigation. Indeed, such kinds of studies are rare in soil research [43]. Studies with repeated sampling over a prolonged period are therefore required [99].

## Supporting information

S1 Table Model summaries for models in Q1. (DOCX)

S2 Table Model summaries for models in Q2. (DOCX)

S3 Table Model summaries for models in Q3. (DOCX)

S4 Table Model summaries for models in Q4. (DOCX)

S5 Table Model summary table for structural equation model in Q5. (DOCX)

S6 Table D-separation tests of variable relationships not included as causal pathways or correlated errors. (DOCX)

S1 Data Deidentified dataset. (XLSX)

S1 Fig (TIF)
