# Supplementary material for: Temporal changes in soil carbon and nitrogen in response to grazing management and vegetation cover in south-eastern Australia
Source: PLoS One. 2026 Feb 6;21(2):e0342006. doi: 10.1371/journal.pone.0342006 (PMC12880676; doi:10.1371/journal.pone.0342006)
Supplement: S1 Table — (DOCX) [file pone.0342006.s001.docx]

***PLOS One -*** *Research Paper*

**Temporal changes in soil carbon and nitrogen in response to grazing management in south-eastern Australia**

**SUPPORTING INFORMATION**

**Table S1. Model summaries for models in Q1.**

| **Model** | **Term** | **Estimate** | **Standard Error** | **P-value** |
| --- | --- | --- | --- | --- |
| Total Carbon (%) | (Intercept) | -3.488 | 0.050 | < 0.001*** |
|  | 2022 | 0.238 | 0.027 | < 0.001*** |
|  | 5-10 cm | -0.809 | 0.025 | < 0.001*** |
| Total Nitrogen (%) | (Intercept) | -6.145 | 0.066 | < 0.001*** |
|  | 2022 | 0.477 | 0.044 | < 0.001*** |
|  | 5-10 cm | -0.804 | 0.037 | < 0.001*** |
| C:N ratio | (Intercept) | 13.903 | 0.474 | < 0.001*** |
|  | 2022 | 0.436 | 0.176 | 0.013* |
|  | 5-10 cm | 1.211 | 0.152 | < 0.001*** |
