# Supplementary material for: Temporal changes in soil carbon and nitrogen in response to grazing management and vegetation cover in south-eastern Australia
Source: PLoS One. 2026 Feb 6;21(2):e0342006. doi: 10.1371/journal.pone.0342006 (PMC12880676; doi:10.1371/journal.pone.0342006)
Supplement: S2 Table — (DOCX) [file pone.0342006.s002.docx]

***PLOS One -*** *Research Paper*

**Temporal changes in soil carbon and nitrogen in response to grazing management in south-eastern Australia**

**SUPPORTING INFORMATION**

**Table S2. Model summaries for models in Q2.**

| **Response** | **Term** | **Estimate** | **Standard Error** | **P-value** |
| --- | --- | --- | --- | --- |
| Total Carbon (%) | (Intercept) | -3.493 | 0.098 | < 0.001*** |
|  | 2022 | 0.480 | 0.058 | < 0.001*** |
|  | Exclusion | -0.093 | 0.109 | 0.391 |
|  | Rotational | 0.034 | 0.101 | 0.738 |
|  | Depth 5-10 cm | -0.812 | 0.024 | < 0.001*** |
|  | 2022 x Exclusion | -0.113 | 0.077 | 0.144 |
|  | 2022 x Rotational | -0.386 | 0.068 | < 0.001*** |
| Total Nitrogen (%) | (Intercept) | -6.575 | 0.154 | < 0.001*** |
|  | 2022 | 1.319 | 0.085 | < 0.001*** |
|  | Exclusion | 0.210 | 0.177 | 0.236 |
|  | Rotational | 0.525 | 0.162 | 0.001** |
|  | Depth 5-10 cm | -0.834 | 0.033 | < 0.001*** |
|  | 2022 x Exclusion | -0.599 | 0.113 | < 0.001*** |
|  | 2022 x Rotational | -1.212 | 0.098 | < 0.001*** |
| C:N ratio | (Intercept) | 14.305 | 0.789 | < 0.001*** |
|  | 2022 | -0.717 | 0.406 | 0.078 |
|  | Exclusion | -0.572 | 0.800 | 0.475 |
|  | Rotational | -0.433 | 0.755 | 0.566 |
|  | Depth 5-10 cm | 1.211 | 0.151 | < 0.001*** |
|  | 2022 x Exclusion | 1.564 | 0.526 | 0.003** |
|  | 2022 x Rotational | 1.338 | 0.469 | 0.004** |
