# Supplementary material for: Temporal changes in soil carbon and nitrogen in response to grazing management and vegetation cover in south-eastern Australia
Source: PLoS One. 2026 Feb 6;21(2):e0342006. doi: 10.1371/journal.pone.0342006 (PMC12880676; doi:10.1371/journal.pone.0342006)
Supplement: S3 Table — (DOCX) [file pone.0342006.s003.docx]

***PLOS One -*** *Research Paper*

**Temporal changes in soil carbon and nitrogen in response to grazing management in south-eastern Australia**

**SUPPORTING INFORMATION**

**Table S3. Model summaries for models in Q3.**

| **Response** | **Term** | **Estimate** | **Standard Error** | **P-value** |
| --- | --- | --- | --- | --- |
| Fractional cover | (Intercept) | 2.752 | 0.088 | < 0.001*** |
|  | 2022 | 0.011 | 0.026 | 0.664 |
|  | Exclusion | 0.241 | 0.094 | 0.011* |
|  | Rotational | 0.131 | 0.089 | 0.142 |
|  | 2022:Exclusion | -0.170 | 0.034 | < 0.001*** |
|  | 2022:Rotational | -0.066 | 0.030 | 0.027* |
| Biomass | (Intercept) | 13.248 | 2.137 | < 0.001*** |
|  | 2022 | -3.190 | 0.654 | < 0.001*** |
|  | Exclusion | 3.951 | 2.225 | 0.076 |
|  | Rotational | 1.671 | 2.109 | 0.428 |
|  | 2022:Exclusion | -0.465 | 0.849 | 0.584 |
|  | 2022:Rotational | -2.994 | 0.755 | < 0.001*** |
| Saplings | (Intercept) | -1.738 | 0.904 | 0.055 |
|  | 2022 | 1.265 | 0.083 | < 0.001*** |
|  | Exclusion | 3.460 | 0.891 | < 0.001*** |
|  | Rotational | 2.046 | 0.852 | 0.016* |
|  | 2022:Exclusion | -0.723 | 0.085 | < 0.001*** |
|  | 2022:Rotational | -0.933 | 0.091 | < 0.001*** |
| Ground cover (exotic) | (Intercept) | 26.668 | 6.757 | < 0.001*** |
|  | 2022 | 10.509 | 1.803 | < 0.001*** |
|  | Exclusion | 0.586 | 6.944 | 0.933 |
|  | Rotational | 2.819 | 6.597 | 0.669 |
|  | 2022:Exclusion | -1.772 | 2.343 | 0.449 |
|  | 2022:Rotational | -7.175 | 2.084 | < 0.001*** |
| Ground cover (native) | (Intercept) | 79.243 | 7.166 | < 0.001*** |
|  | 2022 | -21.458 | 1.927 | < 0.001*** |
|  | Exclusion | 12.129 | 7.755 | 0.118 |
|  | Rotational | 1.587 | 7.284 | 0.828 |
|  | 2022:Exclusion | 6.207 | 2.503 | 0.013* |
|  | 2022:Rotational | 8.108 | 2.227 | < 0.001*** |
| Stems 5-50cm | (Intercept) | 1.374 | 0.793 | 0.083 |
|  | 2022 | -0.339 | 0.045 | < 0.001*** |
|  | Exclusion | 0.666 | 0.841 | 0.428 |
|  | Rotational | -0.390 | 0.800 | 0.626 |
|  | 2022:Exclusion | -0.085 | 0.053 | 0.107 |
|  | 2022:Rotational | 0.332 | 0.054 | < 0.001*** |
| Stems > 50cm | (Intercept) | 0.511 | 0.518 | 0.324 |
|  | 2022 | 0.292 | 0.100 | 0.003** |
|  | Exclusion | -0.722 | 0.615 | 0.240 |
|  | Rotational | -0.662 | 0.565 | 0.241 |
|  | 2022:Exclusion | -0.234 | 0.149 | 0.117 |
|  | 2022:Rotational | -0.115 | 0.124 | 0.354 |
