# Supplementary material for: Temporal changes in soil carbon and nitrogen in response to grazing management and vegetation cover in south-eastern Australia
Source: PLoS One. 2026 Feb 6;21(2):e0342006. doi: 10.1371/journal.pone.0342006 (PMC12880676; doi:10.1371/journal.pone.0342006)
Supplement: S4 Table — (DOCX) [file pone.0342006.s004.docx]

***PLOS One -*** *Research Paper*

**Temporal changes in soil carbon and nitrogen in response to grazing management in south-eastern Australia**

**SUPPORTING INFORMATION**

**Table S4. Model summaries for models in Q4.**

| **Response** | **Term** | **Estimate** | **Standard Error** | **P-value** |
| --- | --- | --- | --- | --- |
| Total Carbon (%) | (Intercept) | -3.481 | 0.047 | < 0.001*** |
|  | Depth 5-10 cm | -0.813 | 0.023 | < 0.001*** |
|  | Stems > 50 cm | -0.024 | 0.030 | 0.430 |
|  | 2022 | 0.180 | 0.030 | < 0.001*** |
|  | Fractional cover | -0.065 | 0.021 | 0.002** |
|  | Stems 5-50 cm | -0.056 | 0.024 | 0.019* |
|  | Ground cover (native) | -0.033 | 0.028 | 0.248 |
|  | Stems > 50 cm x 2022 | 0.222 | 0.026 | < 0.001*** |
|  | 2022 x Ground cover (native) | 0.094 | 0.030 | 0.001** |
| Total Nitrogen (%) | (Intercept) | -6.137 | 0.072 | < 0.001*** |
|  | Depth 5-10 cm | -0.854 | 0.029 | < 0.001*** |
|  | Stems > 50 cm | -0.090 | 0.043 | 0.038* |
|  | Stems 5-50 cm | -0.120 | 0.040 | 0.003** |
|  | 2022 | 0.279 | 0.047 | < 0.001*** |
|  | Fractional cover | -0.005 | 0.045 | 0.915 |
|  | Ground cover (native) | -0.307 | 0.051 | < 0.001*** |
|  | Total_saplings | 0.090 | 0.071 | 0.206 |
|  | BiomassBiomassmean | 0.054 | 0.044 | 0.220 |
|  | Stems > 50 cm x 2022 | 0.631 | 0.040 | < 0.001*** |
|  | 2022 x Fractional cover | -0.151 | 0.060 | 0.012* |
|  | 2022 x Ground cover (native) | 0.378 | 0.045 | < 0.001*** |
|  | 2022 x Total_saplings | -0.175 | 0.046 | < 0.001*** |
|  | 2022 x BiomassBiomassmean | 0.103 | 0.051 | 0.042* |
| C:N ratio | (Intercept) | 13.877 | 0.434 | < 0.001*** |
|  | Ground cover (exotic) | 0.198 | 0.214 | 0.356 |
|  | Total_saplings | 0.590 | 0.222 | 0.008** |
|  | Depth 5-10 cm | 1.214 | 0.147 | < 0.001*** |
|  | Biomassmean | -0.369 | 0.171 | 0.031* |
|  | Stems > 50 cm | 0.224 | 0.225 | 0.318 |
|  | Ground cover (native) | 0.799 | 0.226 | < 0.001*** |
|  | Stems 5-50 cm | 0.427 | 0.161 | 0.008** |
|  | 2022 | 0.576 | 0.218 | 0.008** |
|  | Stems > 50 cm x 2022 | -0.952 | 0.183 | < 0.001*** |
|  | Ground cover (native) x 2022 | -1.006 | 0.230 | < 0.001*** |
|  | Ground cover (exotic) x 2022 | -0.477 | 0.230 | 0.038* |
