# Supplementary material for: Temporal changes in soil carbon and nitrogen in response to grazing management and vegetation cover in south-eastern Australia
Source: PLoS One. 2026 Feb 6;21(2):e0342006. doi: 10.1371/journal.pone.0342006 (PMC12880676; doi:10.1371/journal.pone.0342006)
Supplement: S5 Table — (DOCX) [file pone.0342006.s005.docx]

***PLOS One -*** *Research Paper*

**Temporal changes in soil carbon and nitrogen in response to grazing management in south-eastern Australia**

**SUPPORTING INFORMATION**

**Table S5. Model summary table for structural equation model in Q5.**

| **Response** | **Term** | **Estimate** | **Standard Error** | **Crit.Value** | **P-value** | **Std.Estimate** | **Component** |
| --- | --- | --- | --- | --- | --- | --- | --- |
| Total carbon | Ground cover (exotic) | 0.0246 | 0.0369 | 0.6667 | 0.5050 | 0.0395 | Causal pathway |
| Total carbon | Ground cover (native) | 0.0288 | 0.0358 | 0.8025 | 0.4223 | 0.0462 | Causal pathway |
| Total carbon | Stems > 50 cm | 0.0631 | 0.0358 | 1.7626 | 0.0780 | 0.1014 | Causal pathway |
| Total carbon | Biomass | -0.0131 | 0.0338 | -0.3880 | 0.6980 | -0.0211 | Causal pathway |
| Total carbon | Saplings | -0.0324 | 0.0325 | -0.9965 | 0.3190 | -0.0521 | Causal pathway |
| Total carbon | Stems 5 - 50 cm | -0.0340 | 0.0315 | -1.0774 | 0.2813 | -0.0546 | Causal pathway |
| Total carbon | Fractional cover | -0.0428 | 0.0307 | -1.3969 | 0.1625 | -0.0689 | Causal pathway |
| Total carbon | Rainfall3years | 0.0866 | 0.0893 | 0.9690 | 0.3325 | 0.1392 | Causal pathway |
| Total carbon | Exclusion | -0.0149 | 0.1157 | -0.1291 | 0.8973 | -0.0104 | Causal pathway |
| Total carbon | Rotational | 0.0283 | 0.1034 | 0.2739 | 0.7842 | 0.0224 | Causal pathway |
| Total carbon | 2022 | 0.1437 | 0.2014 | 0.7136 | 0.4755 | 0.1123 | Causal pathway |
| Total carbon | Exclusion (2022) | -0.0774 | 0.1337 | -0.5788 | 0.5627 | -0.0384 | Causal pathway |
| Total carbon | Rotational (2022) | -0.2226 | 0.1191 | -1.8701 | 0.0615 | -0.1447 | Causal pathway |
| Total nitrogen | Ground cover (exotic) | 0.0607 | 0.0450 | 1.3486 | 0.1775 | 0.0826 | Causal pathway |
| Total nitrogen | Ground cover (native) | 0.0394 | 0.0421 | 0.9354 | 0.3496 | 0.0535 | Causal pathway |
| Total nitrogen | Stems > 50 cm | 0.0905 | 0.0424 | 2.1361 | 0.0327* | 0.1231 | Causal pathway |
| Total nitrogen | Biomass | 0.0058 | 0.0395 | 0.1457 | 0.8842 | 0.0078 | Causal pathway |
| Total nitrogen | Saplings | -0.0899 | 0.0393 | -2.2875 | 0.0222* | -0.1223 | Causal pathway |
| Total nitrogen | Stems 5 - 50 cm | -0.0494 | 0.0369 | -1.3372 | 0.1811 | -0.0672 | Causal pathway |
| Total nitrogen | Fractional cover | -0.0412 | 0.0359 | -1.1478 | 0.2511 | -0.0561 | Causal pathway |
| Total nitrogen | Rainfall3years | 0.0244 | 0.1031 | 0.2367 | 0.8129 | 0.0332 | Causal pathway |
| Total nitrogen | Exclusion | 0.0695 | 0.1379 | 0.5044 | 0.6140 | 0.0410 | Causal pathway |
| Total nitrogen | Rotational | 0.1020 | 0.1227 | 0.8314 | 0.4058 | 0.0681 | Causal pathway |
| Total nitrogen | 2022 | 0.4114 | 0.2319 | 1.7740 | 0.0761 | 0.2718 | Causal pathway |
| Total nitrogen | Exclusion (2022) | -0.2344 | 0.1534 | -1.5281 | 0.1265 | -0.0984 | Causal pathway |
| Total nitrogen | Rotational (2022) | -0.3819 | 0.1363 | -2.8012 | 0.0051** | -0.2100 | Causal pathway |
| C:N ratio | Ground cover (exotic) | -0.1668 | 0.2076 | -0.8034 | 0.4218 | -0.0481 | Causal pathway |
| C:N ratio | Ground cover (native) | 0.2214 | 0.2007 | 1.1034 | 0.2699 | 0.0638 | Causal pathway |
| C:N ratio | Stems > 50 cm | -0.1850 | 0.2114 | -0.8753 | 0.3814 | -0.0533 | Causal pathway |
| C:N ratio | Biomass | -0.1913 | 0.1797 | -1.0649 | 0.2869 | -0.0551 | Causal pathway |
| C:N ratio | Saplings | 0.8066 | 0.2246 | 3.5911 | < 0.001*** | 0.2324 | Causal pathway |
| C:N ratio | Stems 5 - 50 cm | 0.2185 | 0.1647 | 1.3269 | 0.1846 | 0.0629 | Causal pathway |
| C:N ratio | Fractional cover | 0.0578 | 0.1636 | 0.3531 | 0.7240 | 0.0166 | Causal pathway |
| C:N ratio | Rainfall3years | 0.4526 | 0.4907 | 0.9223 | 0.3564 | 0.1304 | Causal pathway |
| C:N ratio | Exclusion | -1.3700 | 0.7686 | -1.7825 | 0.0747 | -0.1712 | Causal pathway |
| C:N ratio | Rotational | -0.8292 | 0.6972 | -1.1893 | 0.2343 | -0.1173 | Causal pathway |
| C:N ratio | 2022 | -1.3632 | 1.0454 | -1.3039 | 0.1923 | -0.1908 | Causal pathway |
| C:N ratio | Exclusion (2022) | 1.2255 | 0.5696 | 2.1517 | 0.0314* | 0.1090 | Causal pathway |
| C:N ratio | Rotational (2022) | 1.1193 | 0.5016 | 2.2313 | 0.0257* | 0.1303 | Causal pathway |
| Fractional cover | Exclusion | 0.7650 | 0.3058 | 2.5020 | 0.0123* | 0.3319 | Causal pathway |
| Fractional cover | Rotational | 0.4371 | 0.2896 | 1.5094 | 0.1312 | 0.2145 | Causal pathway |
| Fractional cover | 2022 | -0.5651 | 0.2177 | -2.5964 | 0.0094** | -0.2745 | Causal pathway |
| Fractional cover | Rainfall3years | 0.3389 | 0.1044 | 3.2467 | 0.0012** | 0.3389 | Causal pathway |
| Fractional cover | Exclusion (2022) | -0.4579 | 0.1113 | -4.1157 | < 0.001*** | -0.1413 | Causal pathway |
| Fractional cover | Rotational (2022) | -0.2029 | 0.0989 | -2.0506 | 0.0403* | -0.0820 | Causal pathway |
| Biomass | Exclusion | 0.4741 | 0.2685 | 1.7656 | 0.0775 | 0.2057 | Causal pathway |
| Biomass | Rotational | 0.1986 | 0.2549 | 0.7791 | 0.4359 | 0.0975 | Causal pathway |
| Biomass | 2022 | -0.8721 | 0.2022 | -4.3122 | < 0.001*** | -0.4236 | Causal pathway |
| Biomass | Rainfall3years | 0.2544 | 0.0972 | 2.6158 | 0.0089** | 0.2544 | Causal pathway |
| Biomass | Exclusion (2022) | -0.0482 | 0.1017 | -0.4742 | 0.6353 | -0.0149 | Causal pathway |
| Biomass | Rotational (2022) | -0.3653 | 0.0904 | -4.0399 | < 0.001*** | -0.1477 | Causal pathway |
| Saplings | Exclusion | 0.4363 | 0.3296 | 1.3239 | 0.1855 | 0.1893 | Causal pathway |
| Saplings | Rotational | 0.0687 | 0.2987 | 0.2301 | 0.8180 | 0.0337 | Causal pathway |
| Saplings | 2022 | -0.1719 | 0.0815 | -2.1087 | 0.0350* | -0.0835 | Causal pathway |
| Saplings | Rainfall3years | 0.1300 | 0.0393 | 3.3104 | < 0.001*** | 0.1300 | Causal pathway |
| Saplings | Exclusion (2022) | 0.3443 | 0.0407 | 8.4509 | < 0.001*** | 0.1063 | Causal pathway |
| Saplings | Rotational (2022) | -0.0544 | 0.0362 | -1.5015 | 0.1332 | -0.0220 | Causal pathway |
| Ground cover (exotic) | Exclusion | 0.0191 | 0.2652 | 0.0720 | 0.9426 | 0.0083 | Causal pathway |
| Ground cover (exotic) | Rotational | 0.1036 | 0.2519 | 0.4115 | 0.6807 | 0.0509 | Causal pathway |
| Ground cover (exotic) | 2022 | 0.7224 | 0.1768 | 4.0863 | < 0.001*** | 0.3509 | Causal pathway |
| Ground cover (exotic) | Rainfall3years | -0.1673 | 0.0850 | -1.9692 | 0.0489* | -0.1673 | Causal pathway |
| Ground cover (exotic) | Exclusion (2022) | -0.0729 | 0.0894 | -0.8161 | 0.4145 | -0.0225 | Causal pathway |
| Ground cover (exotic) | Rotational (2022) | -0.2714 | 0.0795 | -3.4147 | < 0.001*** | -0.1097 | Causal pathway |
| Ground cover (native) | Exclusion | 0.4464 | 0.2836 | 1.5737 | 0.1155 | 0.1936 | Causal pathway |
| Ground cover (native) | Rotational | 0.0610 | 0.2665 | 0.2288 | 0.8190 | 0.0299 | Causal pathway |
| Ground cover (native) | 2022 | -0.9230 | 0.1804 | -5.1176 | < 0.001*** | -0.4483 | Causal pathway |
| Ground cover (native) | Rainfall3years | 0.0720 | 0.0866 | 0.8312 | 0.4059 | 0.0720 | Causal pathway |
| Ground cover (native) | Exclusion (2022) | 0.2292 | 0.0916 | 2.5029 | 0.0123* | 0.0707 | Causal pathway |
| Ground cover (native) | Rotational (2022) | 0.2953 | 0.0814 | 3.6267 | < 0.001*** | 0.1194 | Causal pathway |
| Stems 5 - 50 cm | Exclusion | 0.4724 | 0.3041 | 1.5532 | 0.1204 | 0.2049 | Causal pathway |
| Stems 5 - 50 cm | Rotational | -0.0118 | 0.2838 | -0.0416 | 0.9668 | -0.0058 | Causal pathway |
| Stems 5 - 50 cm | 2022 | -0.0987 | 0.2092 | -0.4717 | 0.6371 | -0.0479 | Causal pathway |
| Stems 5 - 50 cm | Rainfall3years | -0.0376 | 0.1001 | -0.3761 | 0.7068 | -0.0376 | Causal pathway |
| Stems 5 - 50 cm | Exclusion (2022) | -0.2437 | 0.1087 | -2.2431 | 0.0249* | -0.0752 | Causal pathway |
| Stems 5 - 50 cm | Rotational (2022) | 0.1694 | 0.0966 | 1.7528 | 0.0796 | 0.0685 | Causal pathway |
| Stems > 50 cm | Exclusion | -0.4073 | 0.2852 | -1.4283 | 0.1532 | -0.1767 | Causal pathway |
| Stems > 50 cm | Rotational | -0.4993 | 0.2695 | -1.8528 | 0.0639 | -0.2450 | Causal pathway |
| Stems > 50 cm | 2022 | 0.0921 | 0.1477 | 0.6235 | 0.5330 | 0.0447 | Causal pathway |
| Stems > 50 cm | Rainfall3years | 0.1837 | 0.0710 | 2.5862 | 0.0097** | 0.1837 | Causal pathway |
| Stems > 50 cm | Exclusion (2022) | -0.3820 | 0.0743 | -5.1396 | < 0.001*** | -0.1179 | Causal pathway |
| Stems > 50 cm | Rotational (2022) | -0.2868 | 0.0661 | -4.3388 | < 0.001*** | -0.1159 | Causal pathway |
| Rainfall | 2022 | 1.9186 | 0.0123 | 155.9702 | < 0.001*** | 0.9320 | Causal pathway |
| Total nitrogen | Total carbon | 0.9512 |  | 86.4316 | < 0.001*** | 0.9512 | Correlated error |
| C:N ratio | Total nitrogen | -0.5765 |  | -19.7782 | < 0.001*** | -0.5765 | Correlated error |
| Fractional cover | Stems 5 - 50 cm | -0.2610 |  | -7.5807 | < 0.001*** | -0.2610 | Correlated error |
| Stems 5 - 50 cm | Saplings | -0.1442 |  | -4.0855 | < 0.001*** | -0.1442 | Correlated error |
| Fractional cover | Biomass | -0.0924 |  | -2.6023 | 0.0047** | -0.0924 | Correlated error |
| Stems 5 - 50 cm | Biomass | -0.1036 |  | -2.9189 | 0.0018** | -0.1036 | Correlated error |
| Saplings | Biomass | 0.0945 |  | 2.6611 | 0.0040** | 0.0945 | Correlated error |
| Fractional cover | Stems > 50 cm | 0.1481 |  | 4.1983 | < 0.001*** | 0.1481 | Correlated error |
| Ground cover (native) | Ground cover (exotic) | -0.1031 |  | -2.9065 | 0.0019** | -0.1031 | Correlated error |
| Saplings | Ground cover (exotic) | -0.1121 |  | -3.1629 | < 0.001*** | -0.1121 | Correlated error |
| Stems 5 - 50 cm | Ground cover (exotic) | -0.0774 |  | -2.1754 | 0.0149* | -0.0774 | Correlated error |
| Fractional cover | Ground cover (exotic) | -0.1036 |  | -2.9212 | 0.0018** | -0.1036 | Correlated error |
| Stems > 50 cm | Ground cover (native) | -0.2414 |  | -6.9734 | < 0.001*** | -0.2414 | Correlated error |
| Biomass | Ground cover (native) | 0.3163 |  | 9.3485 | < 0.001*** | 0.3163 | Correlated error |
| Saplings | Ground cover (native) | -0.3500 |  | -10.4743 | < 0.001*** | -0.3500 | Correlated error |
| Stems 5 - 50 cm | Ground cover (native) | -0.0980 |  | -2.7615 | 0.0029** | -0.0980 | Correlated error |
| Fractional cover | Ground cover (native) | -0.2555 |  | -7.4094 | < 0.001*** | -0.2555 | Correlated error |
| Saplings | Stems > 50 cm | 0.0952 |  | 2.6814 | 0.0037** | 0.0952 | Correlated error |
| Fractional cover | Saplings | 0.3324 |  | 9.8803 | < 0.001*** | 0.3324 | Correlated error |
| C:N ratio | Total carbon | -0.3348 |  | -9.9626 | < 0.001*** | -0.3348 | Correlated error |
| Stems > 50 cm | Ground cover (exotic) | -0.0543 |  | -1.5258 | 0.0637 | -0.0543 | Correlated error |
| Biomass | Ground cover (exotic) | -0.0513 |  | -1.4393 | 0.0752 | -0.0513 | Correlated error |
| Biomass | Stems > 50 cm | 0.0448 |  | 1.2561 | 0.1047 | 0.0448 | Correlated error |
| Stems 5 - 50 cm | Stems > 50 cm | -0.0146 |  | -0.4091 | 0.3413 | -0.0146 | Correlated error |
