# Supplementary material for: Temporal changes in soil carbon and nitrogen in response to grazing management and vegetation cover in south-eastern Australia
Source: PLoS One. 2026 Feb 6;21(2):e0342006. doi: 10.1371/journal.pone.0342006 (PMC12880676; doi:10.1371/journal.pone.0342006)
Supplement: S6 Table — (DOCX) [file pone.0342006.s006.docx]

***PLOS One -*** *Research Paper*

**Temporal changes in soil carbon and nitrogen in response to grazing management in south-eastern Australia**

**SUPPORTING INFORMATION**

**Table S6. D-separation tests of variable relationships not included as causal pathways or correlated errors.**

| Variable 1 | Variable 2 | DF | Critical value | P |
| --- | --- | --- | --- | --- |
| Rainfall | Exclusion | 789 | -0.266 | 0.790 |
| Rainfall | Rotational | 789 | 0.235 | 0.815 |
